# An Eye Tracking Investigation of Pain Decoding Based on Older and Younger Adults’ Facial Expressions

**DOI:** 10.1007/s10919-020-00344-0

**Published:** 2020-10-11

**Authors:** Rhonda J. N. Stopyn, Thomas Hadjistavropoulos, Jeff Loucks

**Affiliations:** Department of Psychology, University of Regina, Regina, SK S4S 0A2 Canada

**Keywords:** Pain communication, Dementia, Eye tracking, Older adults, Facial expressions

## Abstract

Nonverbal pain cues such as facial expressions, are useful in the systematic assessment of pain in people with dementia who have severe limitations in their ability to communicate. Nonetheless, the extent to which observers rely on specific pain-related facial responses (e.g., eye movements, frowning) when judging pain remains unclear. Observers viewed three types of videos of patients expressing pain (younger patients, older patients without dementia, older patients with dementia) while wearing an eye tracker device that recorded their viewing behaviors. They provided pain ratings for each patient in the videos. These observers assigned higher pain ratings to older adults compared to younger adults and the highest pain ratings to patients with dementia. Pain ratings assigned to younger adults showed greater correspondence to objectively coded facial reactions compared to older adults. The correspondence of observer ratings was not affected by the cognitive status of target patients as there were no differences between the ratings assigned to older adults with and without dementia. Observers’ percentage of total dwell time (amount of time that an observer glances or fixates within a defined visual area of interest) across specific facial areas did not predict the correspondence of observers’ pain ratings to objective coding of facial responses. Our results demonstrate that patient characteristics such as age and cognitive status impact the pain decoding process by observers when viewing facial expressions of pain in others.

## Introduction

Observers tend to decode another’s pain experience by interpreting verbal and nonverbal cues (Hadjistavropoulos et al. 2011; Prkachin and Craig 1995). In older adults with dementia, who have limited ability to communicate verbally, observation of nonverbal behaviors is paramount (Browne et al. 2019). Nonetheless, observer and patient characteristics such as age, cognitive status, and observer beliefs can influence the pain decoding process and may confound accurate interpretations (e.g., Hadjistavropoulos et al. 2000a, 2014; Hunter et al. 2013). Our goal was to examine the way in which observers decode nonverbal expressive behaviors of pain in older adults with and without dementia while using eye tracker technology.

Pain in dementia is not only prevalent but is often underreported and undertreated (Achterberg et al. 2013; Corbett et al. 2012; Horgas and Miller 2008; Horgas et al. 2007). While self-report of pain is seen as the gold standard, the ability to report pain deteriorates as dementia progresses (Hobson 2008). Nonverbal pain cues, such as facial expressions, can facilitate valid pain assessments as these cues tend to be relatively preserved even after linguistic and cognitive abilities deteriorate (Hadjistavropoulos and Craig 2002; Hadjistavropoulos et al. 2011; Lints-Martindale et al. 2012). Facial pain expressions consist of distinct patterns of basic muscle movements (Simon et al. 2008). Several facial movements have been proposed to be directly associated with pain expression including: brow lowering, orbit tightening, levator contraction, and eye closure (Prkachin 1992), as demonstrated in numerous studies (e.g., Ashraf et al. 2009; Gallant and Hadjistavropoulos 2017; Hadjistavropoulos et al. 2018; Prkachin 2005; Prkachin and Solomon 2008; Rash et al. 2019; Rocha et al. 2003).

Age-related changes (i.e., wrinkling) in older faces could affect the interpretation of facial expressions by observers such that pain-related cues may be more difficult to discern and decode making pain judgments harder to evaluate compared when observing younger faces (Borod et al. 2004; Ebner and Johnson 2009; Hess et al. 2012; Malatesta et al. 1987; Murphy et al. 2010; Riediger et al. 2011). Moreover, patients with dementia may also tend to react with more vigor to painful stimulation (Hadjistavropoulos et al. 2000b; Kunz et al. 2007). While patterns of facial pain expressions are quite consistent across the lifespan (Williams 2002), observers tend to perceive older adults as experiencing more pain than younger adults even when pain expressiveness is comparable (Hadjistavropoulos et al. 2000a; Matheson 1997). This observer tendency can have a negative effect on the accuracy of pain assessment (Hadjistavropoulos et al. 2014). Perhaps importantly, given that Hess et al. (2012) showed that facial expressions of older people may have less signal clarity than younger people’s expressions, it would be expected that observers’ ratings of pain, might correspond less to fine grained objective coding of facial expressions of older as compared to younger adults.

We aimed to investigate observer ratings of pain as a function of patient dementia status and observer viewing behavior. We considered that gender may also play a role in observer pain judgments (Gagnon et al. 2017), given findings that females tend to be evaluated as experiencing more pain compared to males (Hadjistavropoulos et al. 1996; Lautenbacher et al. 2013; Robinson and Wise 2003; Vigil and Coulombe 2011). Observer pain judgments have also been found to be more accurate for female than for male target patients (Lautenbacher et al. 2013). Nonetheless, there are inconsistencies in the literature concerning the role of target patient gender on observers’ pain judgments (Schafer et al. 2016). Gender stereotypes that presume that women are more sensitive and less tolerant of pain (Robinson et al. 2001; Wandner et al. 2012), may also play a role.

Although facial pain expressions are critical in assessment, caregivers often make pain judgments that do not necessarily correspond to nonverbal pain cues (Eritz and Hadjistavropoulos 2011; Lautenbacher et al. 2013). The purpose of this investigation was to determine to which nonverbal pain behaviors are observers looking at the most and how these cues are used to make pain inferences as a function of patient age and dementia status. To accomplish this goal, we used eye tracking technology to examine observers’ viewing behavior as a function of facial pain expressions in others (Duchowski 2007).

Priebe et al. (2015) and Vervoort et al. (2013) investigated observer viewing behavior directed to facial displays of pain using eye tracking technology. However, these studies used static photographs of pain faces instead of videos. These studies also investigated viewing behaviors of pain faces versus neutral faces (as well as faces depicting other emotional expressions) and established that observers’ visual attention decreased over time when monitoring pain faces compared to neutral faces. Our investigation builds on the previous research by incorporating stimulus videos of pain faces and by examining specific facial areas of interest rather than overall viewing behavior toward painful versus neutral facial expressions.

Through identifying the distribution and total duration of time an observer glances or visually fixates across specific facial areas, it is possible to determine which facial area of interest is most captivating when interpreting another’s pain and whether these areas (e.g., eyes) incorporate key components of pain expressiveness (Duchowski 2007). We were interested in determining whether observers’ visual behavior varied as a function of patient age and dementia status.

It was hypothesized, based on the aforementioned literature, that:

I. Observers would provide higher pain ratings for older adults than for younger persons.
II. Observers’ estimates of older adults’ pain would correspond less to systematically coded facial responses than estimates of younger adults’ pain.
III. Observer pain ratings would show greater correspondence to objective coding of facial expressions when observers look at more nonverbal pain cues.

Patient gender effects were explored. Finally, observer ratings as a function of patient dementia status were also investigated.

## Method

### Observers

Power analyses, assuming alpha = .05 and power level = .80, determined 158 observers would provide more than adequate power to detect small to medium effects (*f *= 0.15) for our analyses. Observers were 164 undergraduate students (128 females and 36 males) with a mean age of 21 years (*SD* = 5). One hundred and thirty-two of our observers reported having close contact with an older adult ranging from daily to less than monthly contact. Two additional observers were not included in the final sample due to technical difficulties with the eye tracker device. Observers were primarily recruited from the department of psychology’s subject pool. An effort was made to recruit comparable numbers of male and female observers. Observers received either course credit or monetary compensation ($10) for participation and provided informed consent as approved by our Institutional Ethics Review Board.

### Stimulus Materials

The videos of younger adult patients and of older adult patients were selected from a larger pool of videos used in previous research (Hadjistavropoulos et al. 2018; Hampton et al. 2018). The selection process was as follows: five videos of male patients with dementia were selected randomly from the larger pool of males with dementia and five videos of female patients with dementia were selected randomly from the larger pool of females with dementia. All patients with dementia were 65 years of age or older. A similar process was followed for older patients without dementia (at least 65 years of age) and for younger adults (23–39 years of age), resulting in a total of 30 videos (10 per experimental condition). All selected videos were cropped and edited into a 30 s segment without audio showing the peak facial pain expression of the patient. The cropped videos only showed the faces of the patients in order to minimize any extraneous information that could bias observers.

In order to evaluate the facial cues of pain in the stimulus videos, we employed the facial action coding system (FACS; Ekman et al. 2002), an objective anatomically-based system that evaluates discrete facial muscle movements, action units (AUs). The FACS has been applied extensively in research on pain expression and determined to be reliable and valid for assessing nonverbal pain behavior, such as facial expressions of pain (Craig et al. 2011). Research suggests that FACS can be used in the assessment of pain in both younger and older adults regardless of the presence of dementia (e.g., Lints-Martindale et al. 2007). We used a validated FACS-based scoring approach (Prkachin 1992; Prkachin and Solomon 2008) that considers the AUs that have been shown in numerous studies to be the most consistently involved in facial pain expressions which include: brow lowering (AU4), cheek raising/lid compression (AU6), lid tightening (AU7), nose wrinkling (AU9), upper lip raising (AU10), and eye closure (AU43). Trained coders watched recordings of facial expressions, and coded the intensity of four actions. Because cheek raising and lid tightening involve actions that are variants of the same underlying muscle group (orbicularis oculi), coders are trained to reduce this action to a single intensity code based on the maximal intensity of the action. This is designated orbit tightening. For the same reason, a single intensity code is rendered for the actions nose-wrinkling and upper-lip raising, yielding a single intensity code for “levator contraction.” Brow lowering (AU 4) receives its own intensity code. The codes for brow lowering, orbit tightening, and levator contraction vary from 0 (*no facial action*) to 5 (*maximal action*). Eyelid closure is coded as absent (0) or present (1). A pain expression index is then constructed by summing the individual action codes. It can vary from 0 to 16 (Prkachin and Solomon 2008). This index has been employed in several studies of pain expression including studies of older adults, that have supported both its reliability and validity (e.g., Ashraf et al. 2009; Gallant and Hadjistavropoulos 2017; Hadjistavropoulos et al. 2018; Prkachin 2005; Prkachin and Solomon 2008; Rash et al. 2019; Rocha et al. 2003). The advantages of this scoring approach are that it focuses on well-established pain-related AUs without the ‘noise’ created by AUs that do not have a consistent relationship with pain thereby reducing assessor burden by decreasing the number of decisions an assessor needs to make.

The independent coding method for these videos, which have also been used in previous research, and the high reliability of that coding were reported in previous studies (Hadjistavropoulos et al. 2018; Hampton et al. 2018). Specifically, reliability values for younger adult videos were *r* = .94 (Hampton et al. 2018), while for older adult videos were *r *= .76 (Hadjistavropoulos et al. 2018). The coded facial cues of pain were used to determine observer viewing behavior and to evaluate the extent to which certain cues were being glanced at for longer durations.

As indicated above, the videos depicted 10 younger and 10 older adults without dementia who were physiotherapy clinic outpatients, as well as 10 older patients with dementia undergoing a safe standardized physiotherapy examination guided by a qualified health professional, which was designed to identify pain (Husebo et al. 2010).^1^ A high definition camera positioned directly overhead recorded all of the videos of the pain patients performing the movements. The final set of the 30 video segments were merged in random order with 15-s intervals between each segment, which allowed for observers to provide pain ratings. To control for order effects, every participant observer viewed a different randomized merged set of the 30 video segments. In total, the final video materials, including the intervals in between video segments, were approximately 23 min in length. The stimulus videos were presented to observers using Experiment Builder (SR Research Ltd.), which is a program designed to integrate with EyeLink eye trackers.

### Equipment

#### Eyelink II

The EyeLink II is a head-mounted video-based eye tracker, which tracks binocular vision and head movement (SR Research Ltd.). The sampling rate of the sensors is 500 Hz and has an accuracy of less than 0.5 degrees with respect to the visual angle. Each observer wore the eye tracker device, which was calibrated to each individual and re-calibrated as necessary during testing. The device tracked and recorded the viewing behaviors of the observers while they watched the stimulus videos in order to discern which specific nonverbal pain cues, contained within facial areas of interest, were being viewed and the corresponding total duration of time the observers gazed at these cues.

More specifically, the faces of the pain patients in the stimulus videos were segmented into areas of interest containing the facial cues of pain including: forehead, brows, eyes, cheeks, nose, mouth, and a non-specified other region. The non-specified other region refers to any other area in the video that did not contain any facial cues of pain. These areas correspond to the scores derived from the FACS-based method described by Prkachin and Solomon (2008), as previously mentioned. The following eye tracking variables were considered:

- “Dwell time” is defined as the amount of time that an observer glances or fixates within a facial area of interest,
- “Total dwell time” refers to an observer’s total dwell time amount between all facial areas of interest,
- “Percentage of total dwell time for each specific area of interest” is defined as the proportion of dwell time an observer spends in one area of interest in relation to all areas of interest.

### Procedure

Eligible participant observers were invited to the lab and were asked to protect the confidentiality of the target patients. Following this, observers were provided with basic education about the nature of dementia (precise script is available from the authors) and completed a demographic questionnaire. Subsequently, each observer had the EyeLink II eye tracker device properly calibrated before testing. All observers took part in each of the three study conditions, which included viewing the 30 stimulus videos. Observers were asked to view the videos while wearing the eye tracker device. They were also asked to provide observational pain intensity ratings for each of the patients in the videos through the use of 100 mm visual analogue scales (VASs) anchored by the polar opposites “*Not intense at all*” to “*Most intense*”. The VAS is a single-item scale that measures subjective experiences such as pain and has been widely used in adult populations (Folstein and Luria 1973; Price et al. 1983; Wilkie et al. 1990). Pain is reported by marking a vertical line perpendicular to the 100 mm scale, which is then measured to obtain the pain rating. The VAS has been shown to be a reliable tool for evaluating pain in others, including those who have limited ability to communicate verbally (Jensen and Karoly 2011; Taddio et al. 2009). Participant observers were provided with a VAS response sheet to record their observational pain ratings once each video ended.

While the observers viewed the video segments, the EyeLink II recorded their viewing behaviors and collected the corresponding data. Specifically, the eye tracker recorded the dwell time of the observer’s attendance to the previously outlined areas of interest containing the facial cues of pain and the percentage of total dwell time among the areas of interest. The eye tracker device was re-calibrated as needed in order to ensure that proper data collection was accomplished.

## Results

### Summary of Analytic Approach

Following examination of the interrater reliability of our FACS coding for our specific stimulus video set, we conducted a 2 (target patient gender: male, female) x 3 (video condition: older adults with dementia, older adults without dementia, young adults) analysis of variance (ANOVA) with FACS-based scores as the dependent variable. The aim of this analysis was to determine if the stimulus videos displayed comparable levels of facial expressiveness across video conditions.

Average observer pain intensity ratings, as well as the percentage of total dwell time among the areas of interest, were calculated for each observer under each video condition. The observer VAS pain intensity ratings and FACS scores were standardized using z-score conversion (*z* = [x − x̄]/s where x̄ is the mean and s the standard deviation) in order to calculate observer rating-FACS correspondence indices. Using FACS scores as an objective index, observer pain rating correspondence scores were calculated (i.e., standardized FACS score − standardized observer rating = correspondence score) and averaged for each observer under each video condition. For example, in a case where a video had a FACS score of 7 and an observer VAS pain intensity rating of 52.60, we first converted the FACS score into a standardized score using the sample mean and standard deviation, (7–6.42)/3.44 = 0.17. A similar procedure was followed for the observer VAS pain intensity rating yielding a standardized value of 0.47. The correspondence score in this case, would be − 0.30 (i.e., 0.17–0.47).

Bonferroni correction was applied to the hypotheses-driven ANOVA analyses to correct for family-wise error rates resulting in an alpha level of .02. In order to test for differences in observer pain ratings as a function of patient age and dementia status, a 2 (target patient gender: male, female) X 3 (video condition: older adults with dementia, older adults without dementia, young adults) within-subjects ANOVA was run. A similar analytic approach was used with observers’ correspondence scores as the dependent variable.

For all ANOVAs, Mauchly’s tests were conducted to test the assumption of sphericity. When this assumption was violated, degrees of freedom were corrected using the Greenhouse–Geisser correction and the Huynh–Feldt correction as appropriate. For all significant main effects, Bonferroni post hoc tests were conducted.

Regression analyses were conducted to examine whether the percentage of observers’ total dwell time among the target areas of interest (brows, cheeks, eyes, forehead, mouth, nose, and other) predicted observer pain rating correspondence scores. For each regression, the following variable was used as a predictor: observers’ percentage of total dwell time for each interest area. One regression was conducted across all video conditions, and additional regressions were conducted specifically within each video condition. In all cases, the regressions were used to predict observer correspondence (to FACS coding). Bonferroni corrections were applied to the four regression analyses resulting in an adjusted alpha level of .01.

Correlation coefficients were calculated to examine the relationship between observers’ visual behavior within the areas of interest containing facial pain cues and the intensity of these pain cues as coded using our FACS-based index. One sample t-tests examined whether observer pain correspondence scores in relation to FACS scores were significantly different from zero (zero scores would be indicative of perfect correspondence). Positive scores that are significantly higher than 0 would indicate underestimation of pain while negative scores would indicate overestimation.

Finally, for exploratory purposes, additional 2 (target patient gender: male, female) X 3 (video condition: older adults with dementia, older adults without dementia, young adults) within-subjects exploratory ANOVAs were conducted to examine differences in observer fixation to facial pain cues as a function of video condition using dwell time within the non-specified other region (which does not contain any facial pain cues), as well as dwell time within the remaining target facial areas of interest as dependent variables. Bonferroni correction was applied to the exploratory ANOVA analyses to correct for family-wise error rates resulting in an alpha level of .007.

### Level of Nonverbal Expressiveness in the Videos

Interrater reliability on our specific set of videos was 0.92 for the global FACS-based score which was used in our analysis (interrater reliability for individual AUs ranged between 0.68 and 0.86). The stimulus video segments all showed comparable levels of facial pain intensity (based on FACS scores) such that there were no significant differences in pain expressions across video conditions or target patient gender. This was determined by the 2 (target gender) X 3 (video condition) within-subjects analysis of variance (ANOVA) that compared the non-verbally displayed pain intensity (as measured by the aforementioned coding approach) across the three video conditions and target patient gender. The main effect of video condition, *F*(2, 8) = .584, *p* = .580, partial *η*^*2*^ = .127, 95% CI [.000, .414], target patient gender, *F*(1, 4) = .035, *p* = .860, partial *η*^*2*^ = .009, 95% CI [.000, .367], and the interaction between video condition and target patient gender, *F*(2, 8) = 2.97, *p* = .108, partial *η*^*2*^ = .426, 95% CI [.000, .651], were not significant. Table 1 shows a summary of the FACS coding of the stimulus videos as a function of video condition and target patient gender.

**Table 1:** Facial action coding system (FACS) scores based on the stimulus videos

Stimulus videos (*N* = 30)	Facial pain cues *M* (SD)				
	Brow lowering	Orbit tightening	Levator contraction	Eye closure	Global pain rating
	(0–5)	(0–5)	(0–5)	(0–1)	(0–16)
Older adults with dementia					
Male	2.8 (1.9)	2.6 (2.2)	2.6 (1.3)	0.4 (0.5)	8.4 (5.4)
Female	2.1 (1.4)	2 (1.1)	1.5 (1.5)	0.2 (0.4)	5.8 (2.4)
Older adults without dementia					
Male	1.2 (1.7)	2.3 (0.9)	1.5 (0.8)	0.2 (0.4)	5.2 (2.2)
Female	2.5 (1.1)	2.9 (1.7)	2.1 (1.4)	0.8 (0.4)	8.3 (3.8)
Younger adults					
Male	1.6 (1.4)	2.6 (1.5)	0.4 (0.7)	0.6 (0.5)	5.2 (1.9)
Female	1.8 (1.8)	2.4 (1.3)	0.8 (1.1)	0.6 (0.5)	5.6 (3.3)

### Observer Pain Ratings

**Fig. 1:**
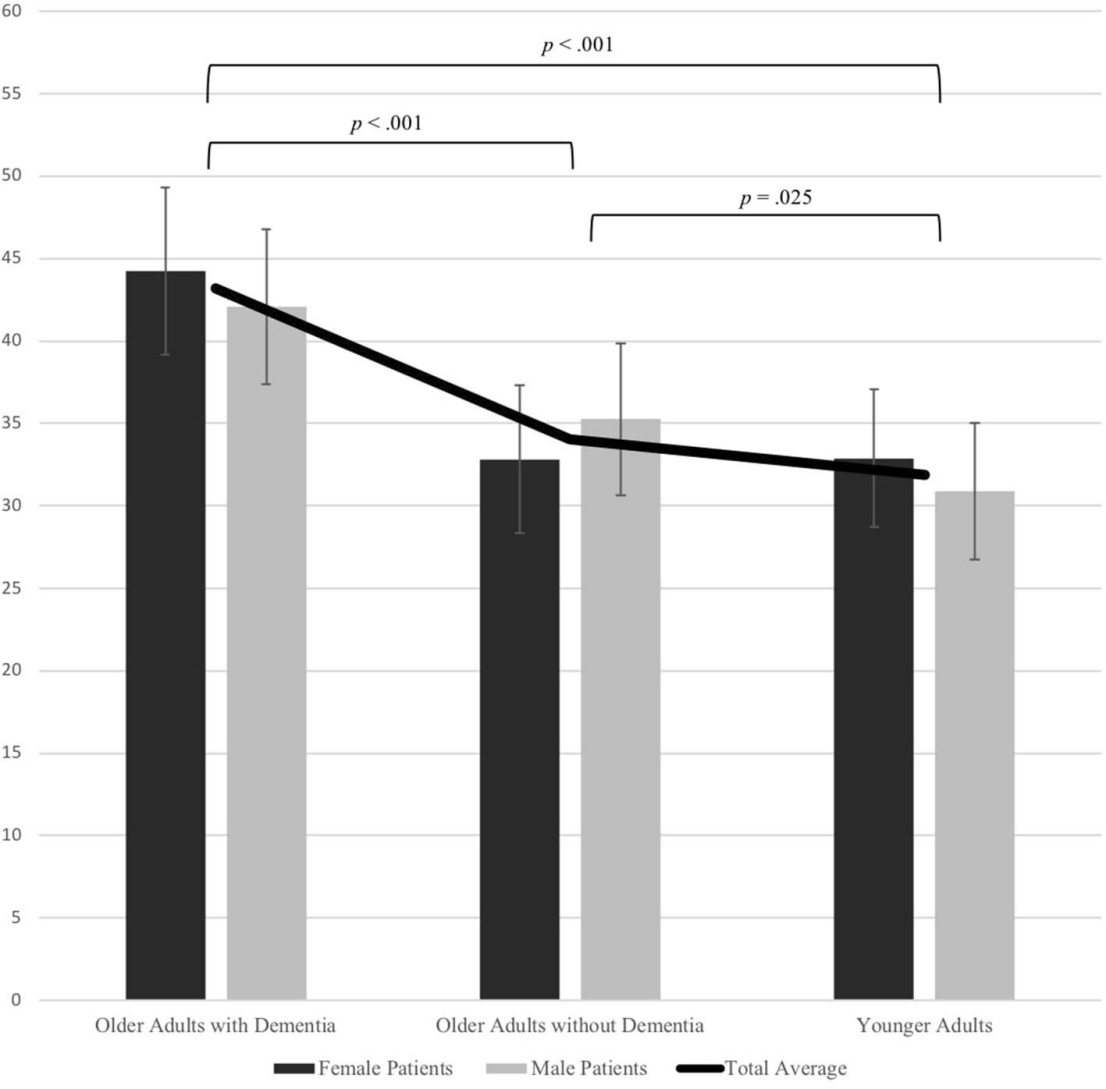
Observers’ pain ratings for video conditions (i.e., older adults with dementia, older adults without dementia, younger adults). The specified significance levels indicate a significant difference between ratings based on older adults with dementia and younger adults, a significant difference between ratings based on older adults with and without dementia and a significant difference in ratings based on older adults with dementia and younger adults

Observer pain ratings are presented in Fig. 1. Consistent with Hypothesis I, a 2 (target patient gender) X 3 (video condition) within-subjects ANOVA demonstrated a significant main effect for video condition, *F*(2, 326) = 106.24, *p* < .001, partial *η*^*2*^ = .395, 95% CI [.314, .462]. There was also a significant interaction between target patient gender and video condition*, F*(1.94, 315.99) = 9.36, *p* < .001, partial *η*^*2*^ = .054, 95% CI [.014, .106]. The main effect of target patient gender was not significant, *F*(1, 163) = 1.25, *p* = .264, partial *η*^*2*^ = .008, 95% CI [.000, .054].

Follow-up pairwise comparisons indicated that observers assigned greater pain intensity ratings to older adults with dementia compared to both older adults without dementia, *MD* = 9.14 (*SE* = .76), *p* < .001, 95% CI [7.29, 10.99], and to younger adults, *MD* = 11.29 (*SE* = .89), *p* < .001, 95% CI [9.13, 13.45]. Pairwise comparisons also demonstrated a significant difference between pain intensity ratings assigned to older adults without dementia and younger adults such that observers rated the pain of older adults without dementia as more intense, *MD* = 2.15 (*SE* = .81), *p* = .025, 95% CI [.21, 4.10]. Additional pairwise comparisons suggested that among patients with dementia, females were assigned greater pain ratings than males, *MD* = 2.12 (*SE* = .92), *p* = .022, 95% CI [.31, 3.93]. Among younger adults, females were also assigned greater pain ratings than males, *MD* = 2.02 (*SE* = .83), *p* = .016, 95% CI [.38, 3.66]. However, among older patients without dementia, males were assigned greater pain ratings than females, *MD* = 2.43 (*SE* = .83), *p* = .004, 95% CI [.79, 4.07].^2^

### Observer Pain Rating Correspondence to Objective Coding of Facial Expressions

**Fig. 2:**
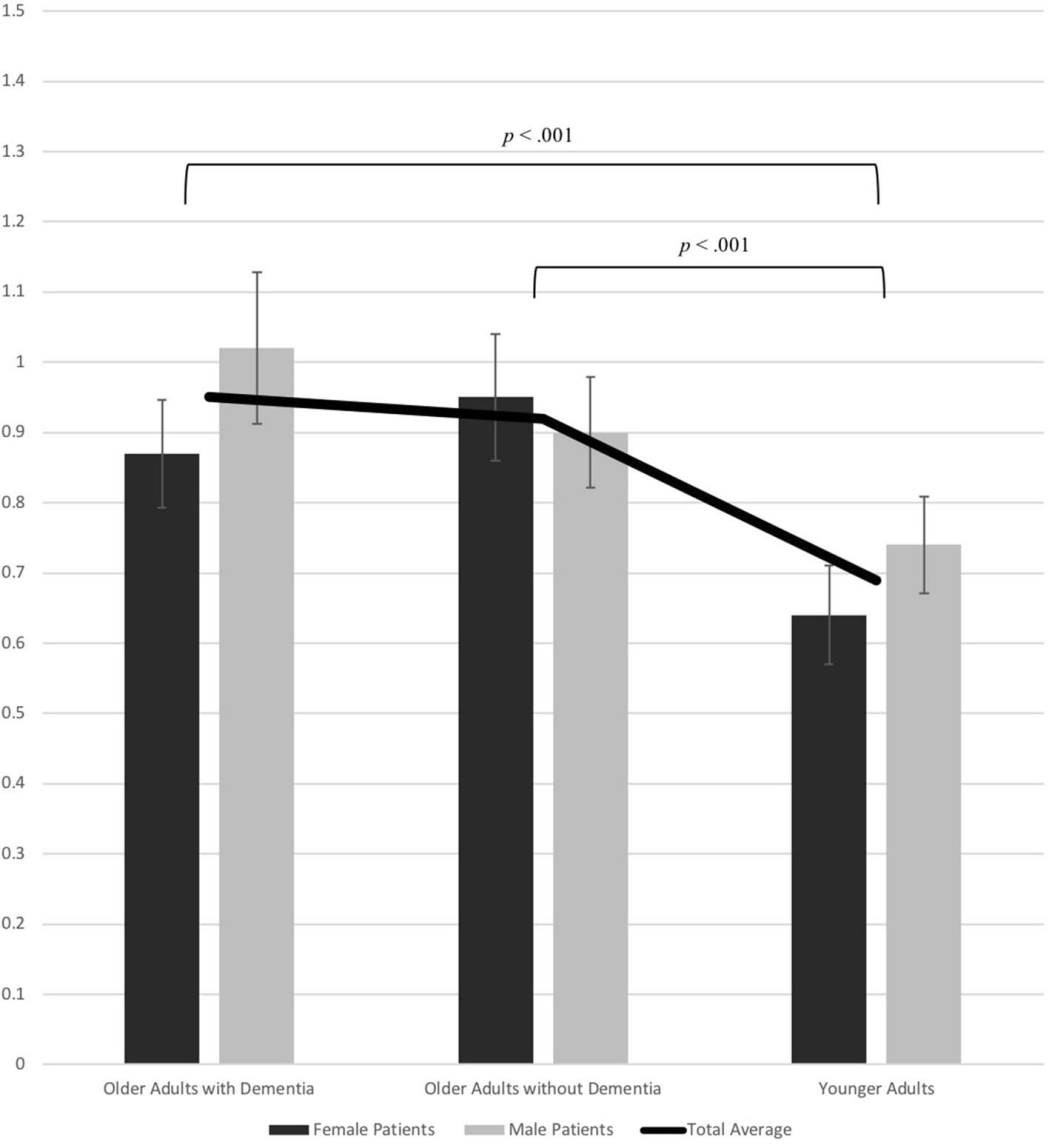
Observers’ correspondence scores for video conditions (i.e., older adults with dementia, older adults without dementia, younger adults). The specified significance levels indicate a significant difference in scores based on older adults with dementia and younger adults and a significant difference in scores based on older adults without dementia and younger adults

Pain rating correspondence scores are presented in Fig. 2. Consistent with Hypothesis II, a 2 (target patient gender) X 3 (video condition) within-subjects ANOVA revealed a main effect for video condition, *F*(1.88, 307.09) = 86.67, *p* < .001, partial *η*^*2*^ = .347, 95% CI [.263, .418]. The main effect of target patient gender was also significant indicating that ratings for female patients showed greater correspondence to objective coding compared to ratings for male patients, *F*(1, 163) = 13.49, *p* < .001, partial *η*^*2*^ = .076, 95% CI [.017, .163]. There was also a significant interaction effect between target patient gender and video condition, *F*(2, 326) = 14.50, *p* < .001, partial *η*^*2*^ = .082, 95% CI [.031, .140].

Follow-up pairwise comparisons indicated that observers’ ratings for younger adults showed greater correspondence compared to ratings for older adults with dementia, *MD* = − 0.26 (*SE* = .02), *p* < .001, 95% CI [−.30, − .21], and older adults without dementia, *MD* = − 0.23 (*SE* = .02), *p *< .001, 95% CI [− .28, − .18]. There were no significant differences between observer correspondence scores for older adults with dementia and older adults without dementia. Follow-up pairwise comparisons also suggested that among patients with dementia, ratings assigned to females corresponded more to objective coding than those assigned to males, *MD* = − 0.15 (*SE* = .03), *p* < .001, 95% CI [− .21, − .09]. Among younger adults, ratings assigned to females corresponded more to objective coding than ratings assigned to males, *MD* = − 0.10 (*SE* = .03), *p* < .001, 95% CI [− .15, − .05]. However, there were no significant differences between males and females among older patients without dementia.

### The Influence of Specific Facial Pain Cues

In order to examine whether observers’ percentage of total dwell time among the areas of interest (brows, cheeks, eyes, forehead, mouth, nose, and other) predicted observer pain rating correspondence scores, multiple linear regression analyses were conducted. Four regression analyses were performed on observer pain rating correspondence scores (i.e., one regression within each video condition and a fourth regression for all observer scores irrespective of condition). Prior to conducting the regression analyses, intercorrelations between dwell time among the areas of interest and correspondence scores were calculated. We adopted a conservative significance level of .01 due to the large number of intercorrelations. None of these exploratory intercorrelations were statistically significant. Regression analyses regarding the full models for overall observer pain rating correspondence, *F*(7, 156) = 0.87, *p* = .530, *R*^*2*^= .038, and the three video conditions, (dementia: *F*[7, 156] = 1.36, *p* = .225, *R*^*2*^= .058, non-dementia: *F*[7, 156] = 1.13, *p* = .348, *R*^*2*^= .048, younger adults: *F*[7, 156] = 1.29, *p* = .260, *R*^*2*^= .055) did not yield significant results either. Contrary to Hypothesis III, observer dwell time proportioned among the target facial areas of interest did not significantly predict observer pain rating correspondence in reference to FACS scores.

### Comparison of FACS Scores and Observer Pain Ratings

One sample t-tests were conducted to test if observer pain rating correspondence scores in relation to FACS scores were significantly different than a score of zero, which would indicate perfect correspondence. Results of the t-tests are presented in Table 2. Observers tended to overestimate the pain of older adults with dementia, underestimate the pain of older adults without dementia, and overall achieved the greatest correspondence to objective coding when assigning pain ratings to younger adults.

**Table 2:** Overestimation/underestimation of pain intensity observer correspondence scores in relation to FACS scores

Video condition	*MD*	*t*	*p*	*Cohen’s d*
Older adults with dementia				
Male	0.32	11.63	.000*	0.91
Female	− 0.53	− 17.82	.000*	1.39
Total average	− 0.11	− 5.40	.000*	0.42
Older adults without dementia				
Male	− 0.30	− 11.17	.000*	0.87
Female	0.73	28.67	.000*	2.24
Total average	0.21	11.93	.000*	0.93
Younger adults				
Male	− 0.11	− 3.67	.000*	0.29
Female	− 0.08	− 3.15	.002*	0.25
Total average	− 0.10	− 4.55	.000*	0.36

A score of 0 would indicate perfect correspondence. Negative mean differences indicate overestimation and positive mean differences indicate underestimation. * *p* < .05. *N* = 164

### Comparison of Observer Dwell Time and Facial Pain Cues

Correlations were also calculated to examine the relationship between observers’ percentage of total dwell time within specific areas of interest containing facial pain cues and the intensity of these facial pain cues as coded using our FACS-based approach. Specifically, the areas of interest examined were: eyes, brows, and nose, as these areas of interest contain the four facial cues most often associated with pain expression. These cues include: brow lowering, orbit tightening, levator contraction, and eye closure. Observers’ fixation to the eyes area of interest was positively correlated with eye closure, *r* = 0.508, *p* < .01. Observers’ fixation to the nose area of interest was negatively correlated with levator contraction, *r* = − 0.371, *p* < .05. Observers’ fixation to the brows area of interest was not significantly correlated with brow lowering, *r* = − 0.007, *p* > .05. Finally, observers’ fixation to the eyes area of interest was not significantly correlated with orbit tightening, *r* = 0.275, *p* > .05.

### Exploratory Analyses of Fixation to Facial Cues

Mean values and SDs are presented in Table 3 for observers’ percentage of total dwell time regarding facial pain cues as a function of video condition. An examination of Table 3 mean scores suggested that across all video conditions observers looked most at the eyes and nose areas of interest. The first 2 (target patient gender) X 3 (video condition) within-subjects ANOVA was conducted to examine effects on observers’ dwell time within the non-specified other area of interest, containing no facial pain cues. The results of this ANOVA revealed a significant main effect of video condition, *F*(1.92, 312.82) = 167.85, *p* < .001, partial *η*^*2*^ = .507, 95% CI [.432, .567]. The main effect of target patient gender, *F*(1, 163) = 0.05, *p* = .828, partial *η*^*2*^ = .000, 95% CI [.000, .024], and the interaction effect between video condition and target patient gender, *F*(1.72, 281.03) = 2.67, *p* = .079, partial *η*^*2*^ = .016, 95% CI [.000, .053], were not significant. Follow-up pairwise comparisons indicated that the area containing no facial pain cues was looked at more when viewing older patients with dementia compared to older patients without dementia, *MD* = 0.06 (*SE* = .00), *p* < .001, 95% CI [.05, .06], and younger patients, *MD* = 0.06 (*SE* = .00), *p* < .001, 95% CI [.05, .07].

**Table 3:** Percentage of total dwell time across all areas of interest

Target facial areas of interest	Video condition *M* (SD)		
	Older adults with dementia	Older adults without dementia	Younger adults
Brows			
Male	9.27 (6.12)	11.98 (7.32)	8.67 (8.20)
Female	11.28 (7.88)	7.95 (6.74)	9.84 (7.34)
Total average	10.28 (6.38)^a^	9.97 (6.14)	9.25 (7.18)^a^
Cheeks			
Male	7.53 (4.98)	1.97 (2.37)	1.91 (2.77)
Female	2.42 (2.67)	2.71 (3.01)	5.59 (4.82)
Total average	4.97 (3.28)^a,b^	2.34 (2.17)^b,c^	3.75 (3.27)^a,c^
Eyes			
Male	30.15 (10.04)	31.23 (13.23)	37.75 (16.38)
Female	27.60 (11.70)	34.00 (13.83)	31.20 (12.24)
Total average	28.87 (10.01)^a,b^	32.61 (12.01)^b,c^	34.47 (13.61)^a,c^
Forehead			
Male	7.41 (8.78)	16.96 (16.58)	5.80 (10.63)
Female	8.69 (11.77)	7.34 (10.24)	9.19 (11.45)
Total average	8.05 (9.89)^b^	12.15 (12.80)^b,c^	7.49 (10.48)^c^
Mouth			
Male	5.68 (5.31)	6.48 (7.70)	7.26 (8.49)
Female	5.83 (6.96)	7.22 (8.22)	8.25 (8.52)
Total average	5.75 (5.59)^a,b^	6.85 (7.42)^b,c^	7.76 (8.07)^a,c^
Nose			
Male	37.68 (13.19)	36.03 (16.46)	46.85 (17.76)
Female	41.06 (15.34)	49.47 (16.42)	41.89 (16.48)
Total average	39.37 (12.85)^a,b^	42.75 (15.23)^b,c^	44.37 (16.44)^a,c^
Other			
Male	9.42 (7.55)	4.87 (7.39)	3.84 (6.08)
Female	10.27 (8.34)	3.85 (5.15)	3.81 (6.19)
Total average	9.84 (6.53)^a,b^	4.36 (5.75)^b^	3.83 (5.39)^a^

The gender listed refers to the patients in the videos. All values are written in percentages. In some instances where there was a slight overlap among the areas of interest, observer’s dwell time was recorded in both applicable areas of interest. *N* = 164

^a^Significant difference between older adults with dementia and younger adults

^b^Significant difference between older adults with dementia and older adults without dementia

^c^Significant difference between older adults without dementia and younger adults

The second 2 (target patient gender) X 3 (video condition) within-subjects ANOVA was conducted to examine effects on observers’ dwell time within the brow area of interest. The results of this ANOVA revealed a significant interaction effect between video condition and target patient gender, *F*(1.92, 313.56) = 39.48, *p* < .001, partial *η*^*2*^ = .195, 95% CI [.120, .267]. As the Bonferroni correction was applied resulting in an alpha level of .007, the main effect of video condition, *F*(1.94, 316.53) = 3.69, *p* = .027, partial *η*^*2*^ = .022, 95% CI [.000, .060], and target patient gender, *F*(1, 163) = 1.29, *p* = .257, partial *η*^*2*^ = .008, 95% CI [.000, .055], were not significant. Follow-up pairwise comparisons suggested the brow area was looked at more when viewing females among patients with dementia, *MD* = 0.02 (*SE* = .01), *p* < .001, 95% CI [.01, .03], and younger adults, *MD* = 0.01 (*SE* = .01), *p* = .014, 95% CI [.00, .02]. Among patients without dementia, the brow area was looked at more when viewing males, *MD* = 0.04 (*SE* = .01), *p* < .001, 95% CI [.03, .05].

The third 2 (target patient gender) X 3 (video condition) within-subjects ANOVA was conducted to examine effects on observers’ dwell time within the cheeks area of interest. The results revealed a significant main effect of video condition, *F*(2, 326) = 77.39, *p* < .001, partial *η*^*2*^ = .322, 95% CI [.241, .392]. There was also a significant interaction effect between video condition and target patient gender, *F*(1.78, 290.24) = 189.13, *p* < .001, partial *η*^*2*^ = .537, 95% CI [.462, .596]. The main effect of target patient gender was not significant, *F*(1, 163) = 1.62, *p* = .205, partial *η*^*2*^ = .010, 95% CI [.000, .060]. Follow-up pairwise comparisons indicated that the cheek area was looked at more when viewing older patients with dementia compared to older patients without dementia, *MD* = 0.03 (*SE* = .00), *p* < .001, 95% CI [.02, .03], and younger patients, *MD* = 0.01 (*SE* = .00), *p* < .001, 95% CI [.01, .02]. The cheek area was also looked at more when viewing younger patients compared to older patients without dementia, *MD* = 0.01 (*SE* = .00), *p* < .001, 95% CI [.01, .02]. Additional pairwise comparisons suggested the cheek area was looked at more when viewing females among patients without dementia, *MD* = 0.01 (*SE* = .00), *p* = .004, 95% CI [.00, .01], and younger adults, *MD* = 0.04 (*SE* = .00), *p* < .001, 95% CI [.03, .04]. Among patients with dementia, the cheek area was looked at more when viewing males, *MD* = 0.05 (*SE* = .00), *p* < .001, 95% CI [.04, .06].

The fourth 2 (target patient gender) X 3 (video condition) within-subjects ANOVA was conducted to examine effects on observers’ dwell time within the eyes area of interest. The results revealed a significant main effect of video condition, *F*(1.87, 304.98) = 43.40, *p* < .001, partial *η*^*2*^ = .210, 95% CI [.133, .284] There was also a significant main effect of target patient gender, *F*(1,163) = 26.83, *p* < .001, partial *η*^*2*^ = .141, 95% CI [.056, .240] with the eye area being looked at more when viewing male patients. Finally, there was also a significant interaction effect between video condition and target patient gender, *F*(1.88, 305.99) = 29.34, *p* < .001, partial *η*^*2*^ = .153, 95% CI [.083, .223]. Follow-up pairwise comparisons indicated that the eye area was looked at less when viewing older patients with dementia compared to older patients without dementia, *MD* = − 0.04 (*SE* = .01), *p* < .001, 95% CI [−.05, −.02], and younger patients, *MD* = − 0.06 (*SE* = .01), *p* < .001, 95% CI [−.07, −.04]. The eye area was also looked at more when viewing younger patients compared to older patients without dementia, *MD* = 0.02 (*SE* = .01), *p* = .023, 95% CI [.00, .04]. Additional pairwise comparisons suggested the eye area was looked at more when viewing males among patients with dementia, *MD* = 0.03 (*SE* = .01), *p* < .001, 95% CI [.01, .04], and younger adults, *MD* = 0.07 (*SE* = .01), *p* < .001, 95% CI [.05, .08]. Among patients without dementia, the eye area was looked at more when viewing females, *MD* = 0.03 (*SE* = .01), *p* = .005, 95% CI [.01, .05].

The fifth 2 (target patient gender) X 3 (video condition) within-subjects ANOVA was conducted to examine effects on observers’ dwell time within the forehead area of interest. The results revealed a significant main effect of video condition, *F*(1.79, 291.99) = 69.60, *p* < .001, partial *η*^*2*^ = .299, 95% CI [.214, .374]. There was also a significant main effect of target patient gender with the forehead area being looked at more when viewing males, *F*(1, 163) = 31.66, *p* < .001, partial *η*^*2*^ = .163, 95% CI [.072, .263]. Finally, there was also a significant interaction effect between video condition and target patient gender, *F*(1.67, 272.75) = 105.29, *p* < .001, partial *η*^*2*^ = .392, 95% CI [.304, .465]. Follow-up pairwise comparisons indicated that the forehead area was looked at more when viewing older patients without dementia compared to older patients with dementia, *MD* = 0.04 (*SE* = .00), *p* < .001, 95% CI [.03, .05], and younger patients, *MD* = 0.05 (*SE* = .01), *p* < .001, 95% CI [.04, .06]. There were no significant differences in dwell time between older patient with dementia and younger adults. Additional pairwise comparisons suggested the forehead area was looked at more when viewing females among patients with dementia, *MD* = 0.01 (*SE* = .01), *p* = .011, 95% CI [.00, .02], and younger adults, *MD* = 0.03 (*SE* = .01), *p* < .001, 95% CI [.02, .05]. Among patients without dementia, the forehead area was looked at more when viewing males, *MD* = 0.10 (*SE* = .01), *p* < .001, 95% CI [.08, .11].

The sixth 2 (target patient gender) X 3 (video condition) within-subjects ANOVA was conducted to examine effects on observers’ dwell time within the mouth area of interest. The results revealed a significant main effect of video condition, *F*(1.78, 289.63) = 25.14, *p* < .001, partial *η*^*2*^ = .134, 95% CI [.067, .204]. There was also a significant main effect of target patient gender, *F*(1, 163) = 9.03, *p* = .003, partial *η*^*2*^ = .052, 95% CI [.006, .131], with the mouth area being looked at more when viewing females. The interaction effect between video condition and target patient gender, *F*(1.83, 298.03) = 0.87, *p* = .411, partial *η*^*2*^ = .005, 95% CI [.000, .029], was not significant. Follow-up pairwise comparisons indicated that the mouth area was looked at more when viewing younger patients compared to older patients with dementia, *MD* = 0.02 (*SE* = .00), *p* < .001, 95% CI [.01, .03], and older patients without dementia, *MD* = 0.01 (*SE* = .00), *p* = .001, 95% CI [.00, .02]. The mouth area was also looked at more when viewing older patients without dementia compared to older patients with dementia, *MD* = 0.01 (*SE* = .00), *p* < .001, 95% CI [.00, .02].

The last 2 (target patient gender) X 3 (video condition) within-subjects ANOVA was conducted to examine effects on observers’ dwell time within the nose area of interest. The results revealed a significant main effect of video condition, *F*(1.87, 304.74) = 30.00, *p* < .001, partial *η*^*2*^ = .155, 95% CI [.086, .226]. There was also a significant main effect of target patient gender, *F*(1, 163) = 78.64, *p* < .001, partial *η*^*2*^ = .325, 95% CI [.213, .425] such that the nose area was looked at more when viewing female patients. Finally, there was a significant interaction effect between video condition and target patient gender, *F*(2, 326) = 91.24, *p* < .001, partial *η*^*2*^ = .359, 95% CI [.278, .428]. Follow-up pairwise comparisons indicated that the nose area was looked at more when viewing younger patients compared to older patients with dementia, *MD* = 0.05 (*SE* = .01), *p* < .001, 95% CI [.03, .07], and older patients without dementia, *MD* = 0.02 (*SE* = .01), *p* = .039, 95% CI [.00, .03]. The nose area was also looked at more when viewing older patients without dementia compared to older patients with dementia, *MD* = 0.03 (*SE* = .01), *p* < .001, 95% CI [.02, .05]. Additional pairwise comparisons suggested the nose area was looked at more when viewing females among patients with dementia, *MD* = 0.03 (*SE* = .01), *p* = .001, 95% CI [.01, .05], and patients without dementia, *MD* = 0.13 (*SE* = .01), *p* < .001, 95% CI [.12, .15]. Among younger adults, the nose area was looked at more when viewing males, *MD* = 0.05 (*SE* = .01), *p* < .001, 95% CI [.04, .06].

## Discussion

Observers’ fixation patterns to facial pain cues as well as pain judgments varied as a function of patient characteristics, including age and cognitive status (i.e., the presence or absence of dementia). That is, older people were viewed as having more pain than younger individuals and older adults with dementia were viewed as having more pain than other patients. Gender differences were identified such that pain inferences differed as a function of target patient gender. These results contribute to our understanding of pain communication (Hadjistavropoulos et al. 2011; Prkachin and Craig 1995) as they further clarify and increase the specificity of the pain decoding process by observers. Results demonstrated that patient characteristics can impact the pain decoding process by observers when viewing others in pain.

A unique and novel contribution of this study was the exploration of the way in which observer’s impressions about pain in older patients with and without dementia related to specific facial cues of pain, using eye tracking technology. While observers tended to look more often at the eyes and nose interest areas across all conditions, they looked less at the facial pain cues of patients with dementia compared to individuals without dementia. Consistent with prior research, results demonstrate that observer judgments regarding other’s pain can be influenced by biases (Hadjistavropoulos et al. 2014, 2011; Hunter et al. 2013).

More specifically, as hypothesized, observers’ pain intensity ratings varied as a function of patient age and dementia status such that greater ratings were assigned to older adults and patients with dementia even though observers were not explicitly told which individuals had dementia. Previous investigations have also suggested observers perceive older adults as experiencing greater pain than younger people (Hadjistavropoulos et al. 2000a; Hampton et al. 2018; Lautenbacher et al. 2013; Matheson 1997). This observer bias could be due to commonly held myths that pain in older age is expected, thus overestimating pain intensity in older adults compared to younger adults (Herr and Mobily 1991; Hofland 1992; Thielke et al. 2012). While observers view older adults as experiencing more intense pain than younger adults, pain in older adults, especially individuals diagnosed with dementia, continues to be underreported and undertreated (Achterberg et al. 2013; Corbett et al. 2012; Horgas and Miller 2008; Horgas et al. 2007).

As hypothesized, observer ratings corresponded more to objectively coded facial reactions when viewing younger adults than older adults. This finding is consistent with prior research (e.g., Horgas and Dunn 2001; Weiner et al. 1999). Observers’ greater rating correspondence for younger adults’ pain may be due to an “own-age bias” (Ebner et al. 2011), although there is research that is inconsistent with this explanation (Lautenbacher et al. 2016). As an alternative explanation, age-related facial changes such as wrinkling could have confounded the correspondence of pain ratings for older adults (Borod et al. 2004; Ebner and Johnson 2009; Hess et al. 2012; Malatesta et al. 1987; Murphy et al. 2010; Riediger et al. 2011). Although age-related facial changes in the controlling of muscle tissue may cause older faces to display unintended blended expressions impacting observer pain ratings (Ebner et al. 2011), in our case facial expressions of older and younger target patients were comparable. Nonetheless, the previous literature and our findings do suggest that facial displays of emotion and pain expressions in older adults, compared to those of younger people, pose decoding challenges for young student observers.

Observer rating correspondence to FACS scores when viewing older adults did not differ between those who had dementia compared to the remaining older adults. Consistent with the literature (Engle et al. 2001; Horgas and Dunn 2001; Scherder and van Manen 2005), findings indicated that observer ratings of pain in both groups of older adults did not show as much correspondence to FACS-based scores as they did for younger patients; pain judgments about younger patients corresponded quite well to the FACS-based scores.

Another possible source of bias on pain judgments was gender. Specifically, patient gender affected observers’ pain ratings, although this varied as a function of target patient characteristics. For example, among older adults with dementia and younger adults, female patients were assigned greater pain ratings compared to males. Among older adults without dementia, however, male patients were assigned greater pain ratings than females. In relation to observer correspondence scores, observer ratings showed the greatest correspondence to FACS coding for female patients among older adults with dementia and younger adults. Patient gender did not affect correspondence scores for older adults without dementia. Past results on the role of patient gender in observer pain inferences have been inconsistent, mirroring the findings of this study (Hadjistavropoulos et al. 1996; Lautenbacher et al. 2013; Pronina and Rule 2014; Riva et al. 2011; Schafer et al. 2016). It has been suggested that gender stereotypes may impact pain judgments such that women are seen as having lower pain tolerance, leading to attributions of greater pain ratings for females (Wandner et al. 2012). Indeed, Robinson and Wise (2003) examined the impact of gender role expectations on pain judgments and found that pain rating variance could be accounted for by such expectations. Further studies are needed to clarify the way in which observer pain inferences vary as a function of patient characteristics, including gender.

In contrast to expectations, the target facial areas of interest were not significantly predictive of observer correspondence scores. In other words, observers do not rely on any one specific area of the face when interpreting the pain experience of others. Previous research that suggested observers do rely on specific facial pain cues used methodologies that were different from our approach. As an example, some research involved blurring parts of the face such that observers did not have access to the entire face when they were exposed to specific facial cues (Roy et al. 2015). Consistent with this study, other investigators found that caregivers of older adults with dementia provided pain ratings of patients unrelated to the pain behaviors, except when there was a high frequency of contact with the patient (Eritz and Hadjistavropoulos 2011). Future examinations are needed of whether time spent with a patient (even among non-caregivers) increases the validity of decoding patient non-verbal pain cues.

It is possible that when observers make pain judgments about others, they tend to take a more holistic view of the pain experience. The findings suggest that our student observers do not rely on a specific facial cue when inferring pain in others, but consider the patient’s face as a whole utilizing multiple facial cues when evaluating pain. Related to this idea, our observers often focused on the nose area (i.e., a central area), possibly because this allows for a holistic perception of the face due to their peripheral vision allowing them to peripherally detect the facial pain cues. That said, correlational results also indicated that observers dwelled more on the eyes when patients displayed the eye closure facial cue, but dwelled less on the nose when the levator contraction pain cue was more intense, possibly due to observers not requiring much time to recognize this intense facial reaction. Observers may also be relying on other contextual factors such as emotional expressions of the face or on their own knowledge regarding pain in others. Of note, spontaneous facial pain expressions conveyed during the physiotherapy examination (which was designed to identify painful areas) allowed for the study to have greater ecological validity compared to studies of experimentally induced pain.

Findings from exploratory analyses examining observer dwell time among the target facial areas of interest containing facial pain cues suggested that observers’ viewing behavior varied as a function of video condition and target patient gender. Future studies are needed to determine the meaning of these results if such findings are replicated. Furthermore, findings indicated that observers looked less at facial pain cues when viewing older adults with dementia compared to older adults without dementia and younger adults. This difference in dwell time duration to facial pain cues could be due to a preference for fixating on faces seen as more physically attractive, as is often the case with younger faces (Foos and Clark 2011; Valuch et al. 2015). These results add to the literature as it is possible observer attitudes may have influenced facial cue fixation behavior.

## Limitations and Future Directions

Prior research has shown that observer characteristics influence the pain decoding process (Hadjistavropoulos et al. 2011). Observer gender may influence observational pain ratings (e.g., Robinson and Wise 2003; Vigil and Coulombe 2011). It has also been suggested that there are age-related changes in emotion and pain recognition when viewing facial expressions such that younger observers may be better at rating others’ pain (Lautenbacher et al. 2016; Ortega and Phillips 2008; Ruffman et al. 2008). Perhaps a more diverse (e.g., with respect to age) sample of participant observers would yield effects based on demographic characteristics, which is something that could not be determined in this study because almost all observers were younger adults. This would be worth pursuing in future research.

Health care professionals and caregivers may infer a patient’s pain through several pain behaviors (Labus et al. 2003). Other contextual cues should be examined, such as body movements in relation to observer pain interpretations. Our observer sample was quite homogenous limiting the generalizability of our findings. As such, further examinations of the relationship between observer gender, observer age, and pain assessments are needed using more diverse samples of participant observers. The amount of experience an observer has with pain has been shown to influence pain inferences reported by health care professionals and non-health professionals, however, all observers in this study were undergraduate students (Hampton et al. 2018; Prkachin et al. 2007). While other studies have suggested only slight improvements in pain recognition and assessment, there is promise that training sessions on facial pain expressions could be beneficial for observers (Kunz and Lautenbacher 2015; Solomon et al. 1997). Future research should further examine the impact of training tools, such as programs designed to teach observers about facial pain expressions, on observer pain judgments of others such as individuals with dementia.

## Conclusion

This investigation provided further understanding of the relationship between nonverbal pain behaviors and observer inferences regarding pain in others, specifically individuals with dementia. As well, differences were found in observers’ viewing behaviors such that observers spent less time gazing at facial pain cues for individuals with dementia. These findings also added to our understanding of influences on observers’ decoding of pain (Hadjistavropoulos et al. 2011; Prkachin and Craig 1995). Observer pain ratings and pain rating correspondence to fine-grained pain coding vary as a function of patient age and cognitive status. While the duration of time an observer spent glancing at specific facial areas did not predict observer pain rating correspondence, more work is needed to determine how observers employ facial pain cues during pain assessments. Future research could aid in facilitating training programs designed to promote more valid pain assessments in others.

## Footnotes

1 Self-report pain ratings were obtained from target patients without dementia. Due to cognitive difficulties patients with dementia were unable to provide pain ratings. The average self-report rating for the physiotherapy examination, based on a 0–10 scale, was 5.08 (*SD *= 2.24). This was significantly higher than the average baseline rating provided by the patients 1.93 (*SD* = 1.73), *t*(19) = 5.29, *p* < .001, *d *= 1.18, 95% CI [.60, 1.75]. This supported the validity of our assumption that the physiotherapy examination increased the pain level of the pain patients.

2 Observers were asked to indicate whether or not they believed each older adult had dementia. On average, observers correctly classified 65% of both groups of videos of older adults as to whether or not the older patients had dementia.
